# Supplementary material for: Strain topological metamaterials and revealing hidden topology in higher-order coordinates
Source: Nat Commun. 2023 Oct 19;14:6633. doi: 10.1038/s41467-023-42321-3 (PMC10587163; doi:10.1038/s41467-023-42321-3)
Supplement: Supplementary file 1 — Supplementary Information [file 41467_2023_42321_MOESM1_ESM.pdf]

Supplementary Information – Strain topological metamaterials and revealing hidden topology in higher-order coordinates

Florian Allein,[1,*] Adamantios Anastasiadis,[2,*] Rajesh Chaunsali,[3,*] Ian Frankel,[4]
Nicholas Boechler,[4] Fotios K. Diakonos,[5] and Georgios Theocharis[2,†]

[1] *Univ. Lille, CNRS, Centrale Lille, Junia, Univ. Polytechnique Hauts-de-France, UMR 8520 - IEMN - Institut d'Electronique de Microélectronique et de Nanotechnologie, F-59000 Lille, France*
[2] *Laboratoire d'Acoustique de l'Université du Mans (LAUM), UMR 6613, Institut d'Acoustique - Graduate School (IA-GS), CNRS, Le Mans Université, France*
[3] *Department of Aerospace Engineering, Indian Institute of Science, Bangalore 560012, India*
[4] *Department of Mechanical and Aerospace Engineering, University of California, San Diego, La Jolla, CA 92093, USA*
[5] *Department of Physics, University of Athens, 15784 Athens, Greece*

CONTENTS

I. Supplementary Note 1 – Mass dimer 2
  A. Displacement coordinates 2
    1. Lack of chiral symmetry in bulk 2
    2. Lack of chiral symmetry in finite chain 2
  B. Strain coordinates 3
    1. Chiral symmetry in bulk 3
    2. Chiral symmetry in finite chain 3
  C. Experimental implementation 4
    1. Design 4
    2. Fabrication and measurements 5

II. Supplementary Note 2 – Mechanical Kitaev chain 6
  A. Displacement coordinates 6
  B. Strain coordinates 7
  C. Free versus fixed boundary conditions for finite chain 8
  D. Experimental implementation 10
    1. Design 10
    2. Fabrication and measurements 12

III. Supplementary Note 3 – Disorder analysis 16
  A. Mass vs. stiffness dimer 16
  B. Mechanical Kitaev chain 18

IV. Supplementary Note 4 – Photonic analogies to mass and spring models 21
  A. Photonic Su-Schrieffer-Heeger (SSH) chain 21
  B. Photonic mass dimer 22

References 24

* These authors contributed equally
† Correspondence and requests for materials should be addressed to G.T. (georgios.theocharis@univ-lemans.fr)

I. SUPPLEMENTARY NOTE 1 – MASS DIMER

A. Displacement coordinates

1. Lack of chiral symmetry in bulk

The equations of motion for this 1D system in the usual displacement coordinates are given by:

$$\begin{aligned} m_1\ddot{u}_{A,n} &= k(u_{B,n} - u_{A,n}) - k(u_{A,n} - u_{B,n-1}) \\ m_2\ddot{u}_{B,n} &= k(u_{A,n+1} - u_{B,n}) - k(u_{B,n} - u_{A,n}), \end{aligned} \tag{1}$$

where the first subscript denotes sublattices A and B within the unit cell and the second subscript denotes the nth unit cell. For the infinite system, we seek the plane wave solutions of the form $\boldsymbol{\psi}_n(t) = \hat{\boldsymbol{u}}(q)e^{i\Omega t - iqn}$ from the equations of motion. This leads to the eigenvalue problem: $D_{u,\text{bulk}}(q)\boldsymbol{u}(q) = \omega^2\boldsymbol{u}(q)$ with

$$D_{u,\text{bulk}}(q) = \frac{1}{(1+P)}\begin{pmatrix} 2 & -(1+e^{-iq}) \\ -P(1+e^{iq}) & 2P \end{pmatrix}. \tag{2}$$

Here $D_{u,\text{bulk}}(q)$ is the bulk dynamical matrix with the subscript u denoting the displacement coordinates, q is the normalized wave number, $\boldsymbol{u}(q) = [u_A(q), u_B(q)]^T$, and $P := m_1/m_2$. Ω is the angular frequency and $\omega = \Omega/\Omega_0$ is the *normalized* frequency with respect to the mid-gap frequency $\Omega_0^2 = k(1/m_1 + 1/m_2)$.

The dynamical matrix is not Hermitian. However, we can redefine $\hat{\boldsymbol{u}} = [\sqrt{P}u_A(q), u_B(q)]^T$ to make the matrix Hermitian of the form:

$$\hat{D}_{u,\text{bulk}}(q) = \frac{1}{(1+P)}\begin{pmatrix} 2 & -\sqrt{P}(1+e^{-iq}) \\ -\sqrt{P}(1+e^{iq}) & 2P \end{pmatrix}. \tag{3}$$

$\hat{D}_{u,\text{bulk}}$ can now be written in terms of the complex Pauli matrices σ_x, σ_y and σ_z such that $\hat{D}_{u,\text{bulk}} = \boldsymbol{I} + d_x\sigma_x + d_y\sigma_y + d_z\sigma_z$ with $d_x = \sqrt{P}(1+\cos q)/(1+P)$, $d_y = \sqrt{P}\sin q/(1+P)$, and $d_z = (1-P)/(1+P)$. It is clear that $\hat{D}_{u,\text{bulk}}$ lacks the chiral symmetry as it does not anti-commute with the chiral operator $\Gamma := \sigma_z$ up to a constant shift in the diagonal as in the standard SSH model [1, 2]. Therefore, it does not have a well-defined winding number for topological characterization.

2. Lack of chiral symmetry in finite chain

The chiral operator for a finite matrix can be defined as the direct sum $\Gamma = \sigma_z \oplus \sigma_z ... \oplus \sigma_z$. A finite mass dimer chain with *even* particles results in a dynamical matrix $\hat{D}_u$ with even dimensions. The matrix is said to be chiral if it obeys: $\Gamma(\hat{D}_u - \boldsymbol{I})\Gamma^{-1} = -(\hat{D}_u - \boldsymbol{I})$. For the case of the mass dimer with *fixed* boundaries ($u_{B,0} = u_{A,N+1} = 0$), the eigenvalue problem is expressed as:

$$\underbrace{\frac{1}{(1+P)}\begin{pmatrix} 2 & -\sqrt{P} & 0 & ... & & 0 \\ -\sqrt{P} & 2P & -\sqrt{P} & ... & & 0 \\ 0 & -\sqrt{P} & 2 & -\sqrt{P} & .. & 0 \\ \vdots & & & \ddots & & \vdots \\ 0 & \dots & & -\sqrt{P} & 2 & -\sqrt{P} \\ 0 & \dots & & & -\sqrt{P} & 2P \end{pmatrix}_{2N\times 2N}}_{\hat{D}_{u,\text{fixed}}} \begin{pmatrix} \hat{u}_{A,1} \\ \hat{u}_{B,1} \\ \hat{u}_{A,2} \\ \vdots \\ \hat{u}_{A,N} \\ \hat{u}_{B,N} \end{pmatrix} = \omega^2 \begin{pmatrix} \hat{u}_{A,1} \\ \hat{u}_{B,1} \\ \hat{u}_{A,2} \\ \vdots \\ \hat{u}_{A,N} \\ \hat{u}_{B,N} \end{pmatrix}, \tag{4}$$

while for the case of *free* boundaries ($u_{B,0} = u_{A,1},\ u_{B,N} = u_{A,N+1}$), it takes the form:

$$\underbrace{\frac{1}{(1+P)}\begin{pmatrix} 1 & -\sqrt{P} & 0 & ... & & 0 \\ -\sqrt{P} & 2P & -\sqrt{P} & ... & & 0 \\ 0 & -\sqrt{P} & 2 & -\sqrt{P} & .. & 0 \\ \vdots & & & \ddots & & \vdots \\ 0 & \dots & & -\sqrt{P} & 2 & -\sqrt{P} \\ 0 & \dots & & & -\sqrt{P} & P \end{pmatrix}_{2N\times 2N}}_{\hat{D}_{u,\text{free}}} \begin{pmatrix} \hat{u}_{A,1} \\ \hat{u}_{B,1} \\ \hat{u}_{A,2} \\ \vdots \\ \hat{u}_{A,N} \\ \hat{u}_{B,N} \end{pmatrix} = \omega^2 \begin{pmatrix} \hat{u}_{A,1} \\ \hat{u}_{B,1} \\ \hat{u}_{A,2} \\ \vdots \\ \hat{u}_{A,N} \\ \hat{u}_{B,N} \end{pmatrix}. \tag{5}$$

Both dynamical matrices in Eq. (4) and Eq. (5) do not anti-commute with Γ after a constant shift in the diagonal. Therefore, it is not obvious if the mass dimer shows any topological features or bulk-boundary correspondence, especially when analyzed in displacement coordinates.

B. Strain coordinates

1. *Chiral symmetry in bulk*

We define the strain coordinates as $s_{A,n} = u_{B,n} - u_{A,n}$ and $s_{B,n} = u_{A,n+1} - u_{B,n}$. We can then rearrange Eq. (1) to get the following equations of motion in strain coordinates:

$$\begin{aligned} \ddot{s}_{A,n} &= \frac{k}{m_2}(s_{B,n} - s_{A,n}) - \frac{k}{m_1}(s_{A,n} - s_{B,n-1}) \\ \ddot{s}_{B,n} &= \frac{k}{m_1}(s_{A,n+1} - s_{B,n}) - \frac{k}{m_2}(s_{B,n} - s_{A,n}). \end{aligned} \tag{6}$$

We again seek the plane wave solutions of the form $\boldsymbol{\psi}_n(t) = \boldsymbol{s}(q)e^{i\Omega t - iqn}$ from the equations of motion to deduce an eigenvalue problem: $D_{s,\text{bulk}}(q)\boldsymbol{s}(q) = \omega^2\boldsymbol{s}(q)$, where $\boldsymbol{s}(q) = [s_A(q), s_B(q)]^T$. The subscript s denotes the strain coordinates. The dynamical matrix takes the form:

$$D_{s,\text{bulk}}(q) = \frac{1}{(1+P)}\begin{pmatrix} 1+P & -(P+e^{-iq}) \\ -(P+e^{iq}) & 1+P \end{pmatrix}. \tag{7}$$

Remarkably, the matrix anti-commutes with σ_z after a constant shift in the diagonal and has a well-defined winding number. The winding is non-trivial for $P < 1$ and trivial for $P > 1$, indicating a topological transition at $P = 1$. The dynamical matrix now resembles the stiffness dimer [2] – the finite-frequency counterpart of the standard SSH model. Therefore, we conclude that by changing the coordinate system in our system, hidden topology is revealed in bulk.

2. *Chiral symmetry in finite chain*

We can quickly check that a finite chain in strain coordinates, with an even number of elements, preserves chiral symmetry for the following boundary conditions: $s_{B,0} = s_{A,N+1} = 0$. In other words, $\Gamma(D_s - \boldsymbol{I})\Gamma^{-1} = -(D_s - \boldsymbol{I})$. Such boundary conditions in strain coordinates resemble a chain with *free* boundaries, i.e., $u_{A,N+1} - u_{B,N} = 0$. The eigenvalue problem reads as follows:

$$\underbrace{\frac{1}{(1+P)}\begin{pmatrix} 1+P & -P & 0 & ... & & 0 \\ -P & 1+P & -1 & ... & & 0 \\ 0 & -1 & 1+P & -P & .. & 0 \\ \vdots & & & \ddots & & \vdots \\ 0 & \dots & & -1 & 1+P & -P \\ 0 & \dots & & & -P & 1+P \end{pmatrix}_{2N\times 2N}}_{D_{s,\text{free}}} \begin{pmatrix} s_{A,1} \\ s_{B,1} \\ s_{A,2} \\ \vdots \\ s_{A,N} \\ s_{B,N} \end{pmatrix} = \omega^2 \begin{pmatrix} s_{A,1} \\ s_{B,1} \\ s_{A,2} \\ \vdots \\ s_{A,N} \\ s_{B,N} \end{pmatrix}, \tag{8}$$

Therefore, it is interesting that strain coordinates reveal the chiral symmetry of a finite chain with *free* boundaries, which was absent in the displacement coordinates discussed earlier. However, there are two crucial points. First, the finite dynamical matrix in strain coordinates should have an *even* number of entries, which means the corresponding lattice in displacement coordinates must have an *odd* number of particles. Second, an odd number of particles means an *odd* number of eigenvalues in the displacement coordinates. For a chain with free boundaries, this includes a zero-frequency rigid body mode. This is naturally excluded when dealing with strain coordinates. Therefore, the remaining *even* number of eigenvalues show symmetry around the midgap frequency due to chiral symmetry, and we can witness the emergence of a topological edge state due to the bulk-boundary correspondence.

Furthermore, we check that in the case of *fixed* boundaries (equivalent to free boundaries in strain coordinates), the eigenvalue problem reads:

$$\underbrace{\frac{1}{(1+P)}\begin{pmatrix} P & -P & 0 & ... & & 0 \\ -P & 1+P & -1 & ... & & 0 \\ 0 & -1 & 1+P & -P & .. & 0 \\ \vdots & & & \ddots & & \vdots \\ 0 & \dots & & -1 & 1+P & -P \\ 0 & \dots & & & -P & 1 \end{pmatrix}_{2N\times 2N}}_{D_{s,\mathrm{fixed}}} \begin{pmatrix} s_{A,1} \\ s_{B,1} \\ s_{A,2} \\ \vdots \\ s_{A,N} \\ s_{B,N} \end{pmatrix} = \omega^2 \begin{pmatrix} s_{A,1} \\ s_{B,1} \\ s_{A,2} \\ \vdots \\ s_{A,N} \\ s_{B,N} \end{pmatrix}. \tag{9}$$

It is clear that the dynamical matrix loses its chirality for the fixed boundaries, and therefore, there is no bulk-boundary correspondence for a mass dimer with fixed boundaries.

C. Experimental implementation

1. *Design*

Beam modeling. To derive the structural stiffness of the beams, we consider a mechanical system consisting of two large cuboid blocks, playing the role of rigid particles, linked by one cylindrical rod (the beam), playing the role of the structural stiffness [see Supplementary Fig. 1]. The model assumes Euler-Bernoulli beam theory and linear (small) deformations. For the case of the mass dimer system, we consider only a translational degree of freedom, namely the normal interaction between each particle. In our model, normal interaction induces only particle translation along the direction passing through their centers. Normal stiffness (K_n) can be determined by analyzing a cantilever beam loaded on its end and comparing the force applied (F) to the elongation (δ). Thus, the stiffness is defined as $K_n = F/\delta = EA/L$ where L is the beam length, E is Young's modulus, and A is the beam cross-section area.

Mass modeling. The unit-cell of the mechanical mass dimer chain is composed of two large (and different) cuboids (the particles) and three cylinders (the beams) as depicted in Supplementary Fig. 1. The total mass m of the unit-cell is defined as $m = m_1 + m_2 + 2m_b$ where m_1 and m_2 refer to the mass of the two particles and m_b to the mass of the beam. The mass of the particle is expressed as $m_i = D_i^3\rho$, where $i = 1, 2$ and the mass of one beam is $m_b = \pi r_b^2 L\rho$ where r_b is the beam radius and ρ is the density.

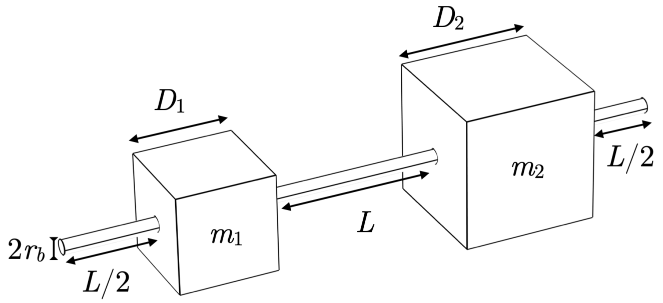

Supplementary Figure 1 | Mechanical mass dimer unit cell.

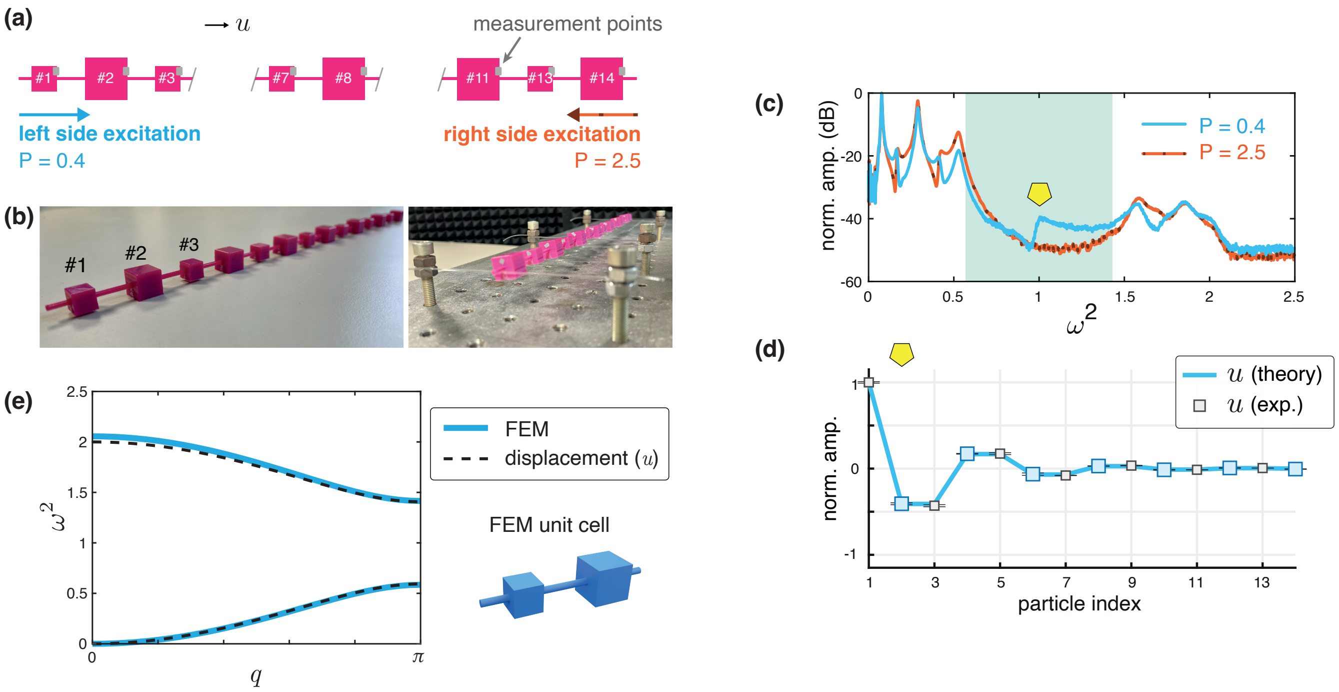

Supplementary Figure 2 | Experimental observation of edge states in the mechanical mass dimer. **(a)** Schematic of the experimental setup. **(b)** Photograph of the mass-dimer chain and experimental setup suspended on thin strings. **(c)** Measured frequency response at particle #8 when the chain is excited from the left side (small mass end) or the right side (large mass end). The blue area corresponds to the band gap. **(d)** Measured amplitudes of the edge state (displaced in displacement coordinates) localized at the boundary ending with small mass and $P = 0.4$. Error bars denote variation (one standard deviation from averaged value) in experimental measurements. **(e)** Dispersion curve calculated from the theoretical model (dashed line) and FEM calculations (solid line).

2. *Fabrication and measurements*

Additive manufacturing of chain. The mechanical mass-dimer chain comprises 14 masses, i.e., 7 unit cells, and is fabricated through additive manufacturing (Stratasys Objet350 Connex3). Thermoplastic polymer (VERO^{TM}), with the following nominal properties, has been used: density $\rho = 1180$ kg/m^3, Young's modulus $E = 2.74$ GPa, and Poisson ratio $\nu = 0.38$. The manufactured sample is presented in Supplementary Fig. 2**(b)**. The mass ratio, defined as $P = m_1/m_2$, is set to $P = 0.4$. The beam geometrical parameters are $L = 15$ mm, $r_b = 1$ mm, and the cube (masses) side lengths are $D_1 = 8$ mm and $D_2 = D_1/P^{1/3} \approx 10.86$ mm.

For a large chain with a negligible interaction between two boundaries, we expect the emergence of an edge state at its boundary if the chain ends by a small particle ($P < 1$), as dictated by the BBC. As such, a chain starting with a small mass and ending with a large mass should only have only one edge state at the edge starting with the small mass. This configuration has been chosen for the designed sample: the first mass of the chain (#1) is smaller (thus equivalent to $P = 0.4$) while the last mass (#14) is larger (thus equivalent to $P = 2.5$), as depicted in Supplementary Fig. 2**(a)**.

Frequency response measurements. Elastic waves are excited by striking the particle #1 and the particle #14 in separate experiments. The chain is suspended on thin strings; therefore, all particles are considered free. Supplementary Fig. 2**(c)** presents measured frequency response at particle #8. We witness a band gap (highlighted region) and a peak inside it, which appears for a given side of excitation, corresponding to the edge state. The state inside the band gap exists for $P = 0.4$, as theoretically predicted. Furthermore, we reconstruct the mode shape from the experimental data in Supplementary Fig. 2**(d)**. We observe excellent agreement between predictions and experiments, where amplitude decay can be seen as one goes away from the boundary. In addition, we present in Supplementary Fig. 2**(e)** the dispersion curves of the mass dimer unit cell calculated by FEM and compared to the theoretical mass-spring model. Once again, we observe an excellent agreement between the two models, which validates our design.

II. SUPPLEMENTARY NOTE 2 – MECHANICAL KITAEV CHAIN

A. Displacement coordinates

The equations of motion governing the linear dynamics of the mechanical chain considering the transverse-rotational waves are derived using the Lagrangian formalism [3, 4] and are described by the following set of differential equations:

$$
\begin{aligned}
m_n \ddot{u}_n = & K_{S,n}\left(u_{n-1}-u_n\right)-K_{S,n+1}\left(u_n-u_{n+1}\right) \\
& +dK_{S,n}\left(\phi_n+\phi_{n-1}\right)-dK_{S,n+1}\left(\phi_n+\phi_{n+1}\right), \qquad (10a)\\
I_n \ddot{\phi}_n = & dK_{S,n}\left(u_n-u_{n-1}-d\left(\phi_{n-1}+\phi_n\right)\right) \\
& +dK_{S,n+1}\left(u_{n+1}-u_n-d\left(\phi_n+\phi_{n+1}\right)\right) \\
& +d^2K_{B,n}\left(\phi_{n-1}-\phi_n\right)-d^2K_{B,n+1}\left(\phi_n-\phi_{n+1}\right), \qquad (10b)
\end{aligned}
$$

where n is the particle index, m is the mass, I is the moment of inertia, and u_n and ϕ_n are the transverse displacement (along the y-axis) and rotation (around the z-axis), respectively, from the equilibrium position of the nth particle. Substituting the plane wave solutions of the form $\boldsymbol{\psi}_n(t)=\boldsymbol{v}(q)e^{i\Omega t-iqn}$ into the set of Eq. (10) leads to the eigenvalue problem:

$$D_{u,\text{bulk}}(q)\boldsymbol{v}(q)=\omega^2\boldsymbol{v}(q), \qquad (11)$$

where $\boldsymbol{v}(q)=[u(q),\Phi(q)/\sqrt{P}]^T$ is a column eigenvectors with $\Phi(q)/\sqrt{P}=d\phi(q)/\sqrt{P}$ and $P=md^2/I$. The superscript T denotes the transposed vector, and $D_{u,\text{bulk}}$ is the bulk dynamical matrix, where the subscript u denotes the displacement coordinates. Variable q is the normalized wave number in the x-direction, Ω is the angular frequency, and $\omega=\Omega/\Omega_0$ is the *normalized* frequency with $\Omega_0^2=2PK_S/m$. The dynamical matrix $D_{u,\text{bulk}}$ can be read as:

$$D_{u,\text{bulk}}=\frac{1}{2P}\begin{bmatrix}4\sin^2(q/2) & -2i\sqrt{P}\sin(q)\\ 2i\sqrt{P}\sin(q) & 4P\left[\cos^2(q/2)+\eta\sin^2(q/2)\right]\end{bmatrix}, \qquad (12)$$

where $\eta=K_B/K_S$ is the ratio of generalized stiffness (with K_B and K_S the bending and shear stiffness, respectively). Under the condition $\eta=1-(1/P)$, this matrix can be fine-tuned to a new form of the dynamical matrix:

$$\tilde{D}_{u,\text{bulk}}=\frac{1}{2P}\begin{bmatrix}2P+[-2(P-1)-2\cos(q)] & -2i\sqrt{P}\sin(q)\\ 2i\sqrt{P}\sin(q) & 2P+[2(P-1)+2\cos(q)]\end{bmatrix}, \qquad (13)$$

which resembles the BdG Hamiltonian when taking out the constant term $2P$ of the diagonal. Comparing with the BdG Hamiltonian [5], we find that $\Delta\to\sqrt{P}$, $\tau\to 1$, $\mu\to 2(P-1)$. The matrix $\tilde{D}$ can be written in terms of the complex Pauli matrices σ_x, σ_y and σ_z such that:

$$\tilde{D}_{u,\text{bulk}}=\boldsymbol{I}+\frac{1}{2P}\left[\left(2\sqrt{P}\sin(q)\right)\sigma_y+\left(-2\cos(q)-2(P-1)\right)\sigma_z\right]. \qquad (14)$$

We check

$$\sigma_x\left(\tilde{D}_{u,\text{bulk}}-\boldsymbol{I}\right)\sigma_x^T=-\left(\tilde{D}_{u,\text{bulk}}-\boldsymbol{I}\right), \qquad (15)$$

where $\boldsymbol{I}$ is the identity matrix. The Bloch vector lies in a 2D plane; therefore, we look for a winding number and observed that our system is in a trivial phase when $P>2$ ($\eta>1/2$) and a nontrivial phase when $0<P<2$ ($\eta<1/2$) with a topological transition point at $P=2$ ($\eta=1/2$). Supplementary Fig. **3** shows the dispersion diagram for these cases.

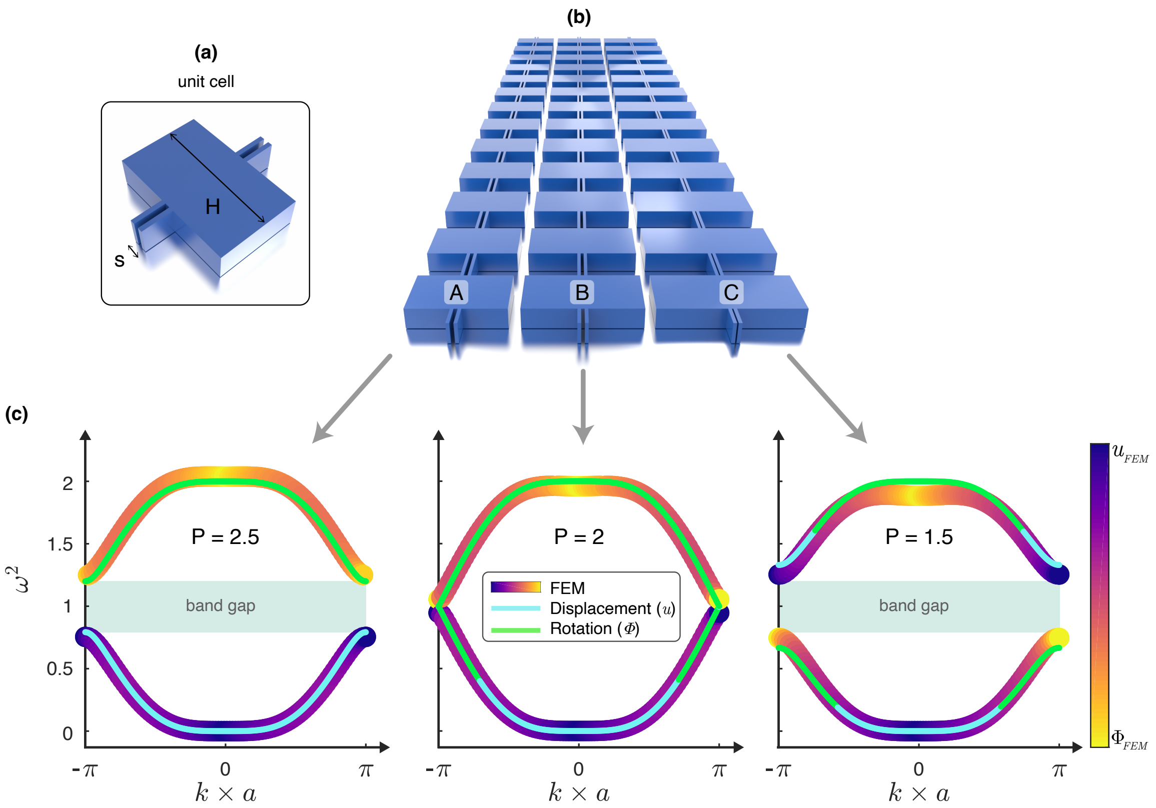

Supplementary Figure 3 | (a-b) Mechanical structures and **(c)** associated dispersion curves from the theoretical model and FEM calculations for several values of P. Notice the band gap closing at $P = 2$.

B. Strain coordinates

We will now demonstrate that a different coordinate system is needed to understand the bulk-edge correspondence for the Free-Free system (discussed in the later next subsection) and to highlight that the edge states, in that case, are also of topological origin. Since we are interested in the in-plane degrees of freedom, which are decoupled from the longitudinal displacements, we will use a formulation based on the shear and bending strains:

$$s_n = u_{n+1} - u_n - d(\phi_{n+1} + \phi_n) \tag{16}$$

$$b_n = d(\phi_{n+1} - \phi_n), \tag{17}$$

where s_n stands for the nth shear strain and b_n stands for the nth bending strain. With this transformation, we shift from the displacement coordinates to the strain coordinates. We already know the equations of motion for transverse displacements and rotations:

$$m\ddot{u}_n = K_S s_n - K_S s_{n-1} \tag{18}$$

$$I\ddot{\phi}_n = d\left(K_S s_n + K_S s_{n-1} + K_B b_n - K_B b_{n-1}\right). \tag{19}$$

We then take the second derivative with respect to time in Eq. (16) and Eq. (17) and substitute the corresponding expressions from Eq. (18) and Eq. (19). The resulting dynamical equations for the strains are:

$$\ddot{s}_n = \frac{K_S}{m}\left(s_{n+1} + s_{n-1} - 2s_n - \frac{md^2}{I}\left(s_{n+1} + s_{n-1} + 2s_n\right) - \frac{md^2}{I}\frac{K_B}{K_S}\left(b_{n+1} - b_{n-1}\right)\right) \tag{20}$$

$$\ddot{b}_n = \frac{K_S}{m}\left(\frac{md^2}{I}(s_{n+1} - s_{n-1}) + \frac{md^2}{I}\frac{K_B}{K_S}(b_{n+1} + b_{n-1} - 2b_n)\right). \tag{21}$$

Next, we assume solutions of the form $\boldsymbol{\psi}_n(t) = \boldsymbol{s}(q)e^{i(\Omega t - qn)}$. We define: $\boldsymbol{s}(q) = [s(q), b(q)/\sqrt{\frac{P}{P-1}}]^T$, where $P = \frac{md^2}{I}$. We thus arrive at the following eigenvalue problem:

$$D_{s,\text{bulk}}(q)\boldsymbol{s}(q) = \omega^2 \boldsymbol{s}(q), \tag{22}$$

where $D_{s,\text{bulk}}$ denotes the "strain" dynamical matrix and ω is the *normalised* frequency Ω/Ω_0 with $\Omega_0^2 = 2PK_s/m$. We define $\eta = K_B/K_S$ and impose $\eta = 1 - 1/P$. The resulting strain dynamical matrix is

$$\tilde{D}_{s,\text{bulk}} = \frac{P-1}{2P}\begin{pmatrix} \frac{2P}{P-1} + \frac{2}{P-1} + 2\cos(q) & -2i\sqrt{\frac{P}{P-1}}\sin(q) \\ 2i\sqrt{\frac{P}{P-1}}\sin(q) & \frac{2P}{P-1} - \frac{2}{P-1} - 2\cos(q) \end{pmatrix}. \tag{23}$$

Note that apart from a constant shift $\frac{2P}{P-1}$ in the diagonal, the matrix possesses particle-hole symmetry. Importantly, this matrix transforms to the displacement dynamical matrix $\tilde{D}_{u,\text{bulk}}$ in Eq. (13) under the following transformation $P \to P/(P-1)$.

C. Free versus fixed boundary conditions for finite chain

In this section, we will present the finite dynamical matrices for the mechanical Kitaev chain in displacement and strain coordinates, $\tilde{D}_u$ and $\tilde{D}_s$ respectively, for both fixed and free boundaries. We will show that in the case of fixed boundaries, $\tilde{D}_u$ preserves the particle-hole symmetry of the bulk dynamical matrix of Eq. (13), and edge states emerge according to BBC. Interestingly, for the case of free boundaries, $\tilde{D}_u$ also exhibits edge states, but for boundaries that break particle-hole symmetry and in the "wrong" parameter regime – the regime where the bulk dynamical matrix of Eq. (13) gives zero winding. Consequently, we show that the finite dynamical matrix on strains $\tilde{D}_s$ preserves the symmetry for the free boundaries, and it follows the BBC given by Eq. (23).

In general, using the basis $\boldsymbol{U} = [u_1, \Phi_1/\sqrt{P}, ..., u_N, \Phi_N/\sqrt{P}]^T$, the $2N \times 2N$ dynamical matrix describing the modes of the mechanical chain has a block tridiagonal form expressed by the $N \times N$ matrix:

$$\mathcal{D}_{bc}(N) = \begin{bmatrix} \mathbf{A_1}_{bc} & \mathbf{B}_{bc} & \mathbf{O} & \dots & \dots & \mathbf{O} \\ \mathbf{B^T}_{bc} & \mathbf{A}_{bc} & \mathbf{B}_{bc} & \mathbf{O} & \dots & \dots \\ \mathbf{O} & \dots & \dots & \dots & \dots & \dots \\ \dots & \dots & \dots & \dots & \dots & \mathbf{O} \\ \dots & \dots & \mathbf{O} & \mathbf{B^T}_{bc} & \mathbf{A}_{bc} & \mathbf{B}_{bc} \\ \mathbf{O} & \dots & \dots & \mathbf{O} & \mathbf{B^T}_{bc} & \mathbf{A_N}_{bc} \end{bmatrix} \tag{24}$$

where $\mathbf{O}$ is the 2×2 zero matrix while $\mathbf{A_1}_{bc}$, $\mathbf{A}_{bc}$, $\mathbf{A_N}_{bc}$, $\mathbf{B}_{bc}$ are also 2×2 matrices having a form which depends on the boundary conditions of the chain. This is indicated by the index "bc". After the normalization described in the previous section and the fixing of the parameter η to the value $\eta = 1 - \frac{1}{P}$, for a chain fixed at both ends we have:

$$\mathbf{B}_{fixed} = \begin{bmatrix} -\frac{1}{2P} & \frac{1}{2\sqrt{P}} \\ -\frac{1}{2\sqrt{P}} & \frac{1}{2P} \end{bmatrix}, \ \mathbf{A_1}_{fixed} = \mathbf{A_N}_{fixed} = \mathbf{A}_{fixed} = \begin{bmatrix} \frac{1}{P} & 0 \\ 0 & 2 - \frac{1}{P} \end{bmatrix}. \tag{25}$$

When both ends of the chain are free we find:

$$\mathbf{B}_{free} = \mathbf{B}_{fixed} \quad ; \quad \mathbf{A}_{free} = \mathbf{A}_{fixed} \quad ; \mathbf{A_1}_{free} = \begin{bmatrix} \frac{1}{2P} & \frac{1}{2\sqrt{P}} \\ \frac{1}{2\sqrt{P}} & 1 - \frac{1}{2P} \end{bmatrix} \quad ; \quad \mathbf{A_N}_{free} = \begin{bmatrix} \frac{1}{2P} & -\frac{1}{2\sqrt{P}} \\ -\frac{1}{2\sqrt{P}} & 1 - \frac{1}{2P} \end{bmatrix}.$$

As is evident, the particle-hole symmetry of the bulk is preserved only for the fixed boundary. As is shown in Supplementary Fig. **4(a)**, the emergence of edge states agrees with the prediction of the bulk winding of the bulk dynamical matrix [Supplementary Fig. **4(b)**].
In the case of free boundaries, the first and last blocks of Eq. (24) take the form Eq. (26). Apparently, these boundaries break the symmetry of the bulk. Interestingly, this dynamical matrix also exhibits edge states but in the anticipated "trivial" parameter regime as is shown in Supplementary Fig. **4(d)**. Do these edge states have a topological origin, or should they be treated just as "defects"?

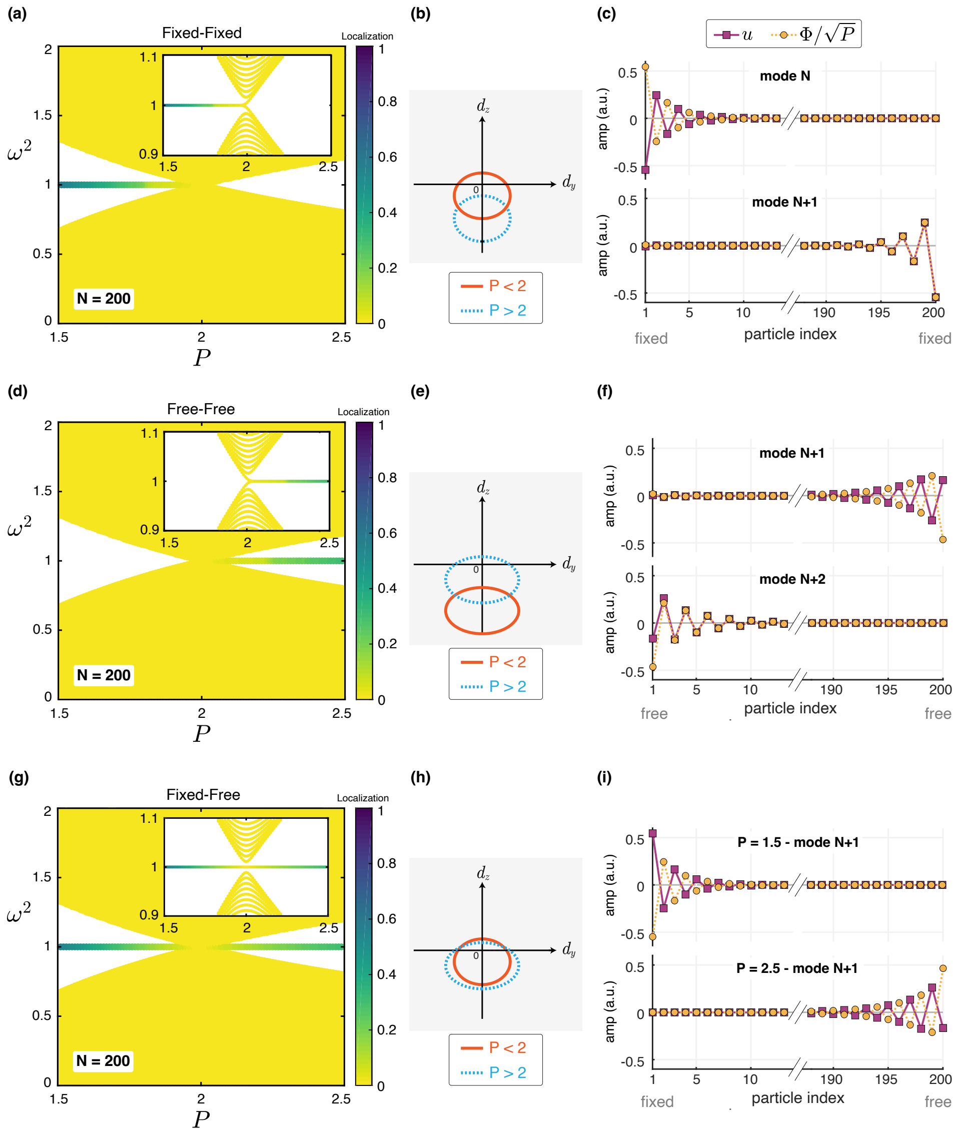

Supplementary Figure 4 | Finite system eigenanalysis for **(a-c)** fixed-fixed, **(d-f)** free-free, and **(g-i)** fixed-free boundary conditions and N = 200 particles. Left column presents the spectra as a function of the P value. The insets show a close-up around the midgap frequency. Middle column shows the winding number of the Bloch vector in d_y-d_z plane. The right column presents the edge modes for the different boundary conditions.

Beginning from Eq. (20) and Eq. (21), one can obtain the finite dynamical matrix for free boundaries on strain coordinates by applying $s|_0 = b|_0 = s|_{N+1} = b|_{N+1} = 0$. The resulting matrix has the same form as Eq. (24) but this time with:

$$\mathbf{B}_{free} = \begin{bmatrix} \frac{1}{2\tilde{P}} & \frac{1}{2\sqrt{\tilde{P}}} \\ -\frac{1}{2\sqrt{\tilde{P}}} & -\frac{1}{2\tilde{P}} \end{bmatrix}, \quad \mathbf{A}_{free} = \mathbf{A}_{1,free} = \mathbf{A}_{N,free} = \begin{bmatrix} 2-\frac{1}{\tilde{P}} & 0 \\ 0 & \frac{1}{\tilde{P}} \end{bmatrix}, \tag{26}$$

where $\tilde{P} = P/(P-1))$. As is evident, the free boundaries preserve the symmetry in this coordinate system, but BBC should now follow the predictions of Eq. (23). Comparing Supplementary Fig. **4(d)** with the bulk winding of Eq. (23) as exhibited in Supplementary Fig. **4(e)** we see that BBC is indeed restored and topological phases are interchanged since Eq. (23) has the same structure as Eq. (13) under the transformed system parameter $P \to P/(P-1)$. The fixed point of this transformation is $P = 2$, which coincides with the gap closing of the spectrum.

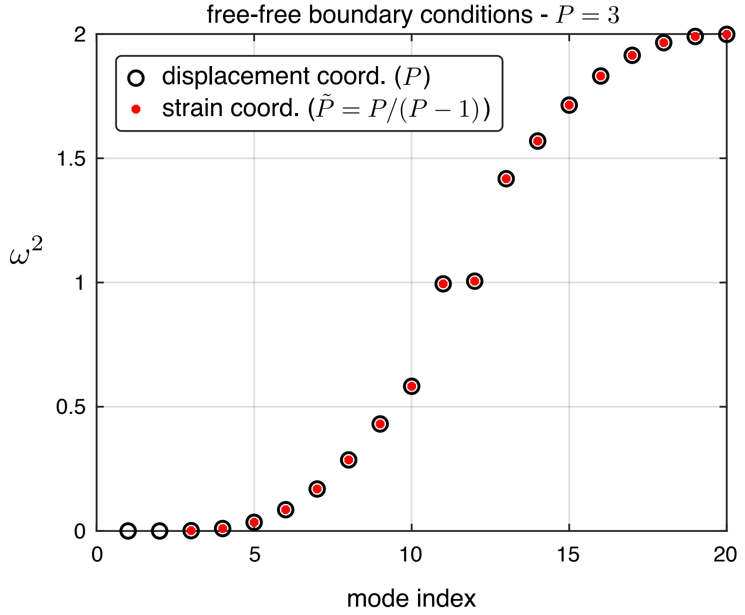

Supplementary Figure 5 | Spectrum comparison of a free-free chain of $N = 10$ particles with parameter $P = 3$ in displacement coordinates to a free-free chain in strain coordinates with parameter $\tilde{P} = P/(P-1)$ and an equal number of particles. Note that two cases have identical eigenfrequencies except two zero modes for the chain described in displacement coordinates.

We observe that applying strain coordinates reveals the hidden topological nature of the edge state appearing for the free boundary. Combining the aforementioned results, we should expect omnipresent edge states for this mechanical Kitaev chain with a fixed-free boundary as is shown in Supplementary Fig. **4(g)**. The reason for this is that one boundary will behave according to BBC for fixed boundaries and the other boundary according to BBC for free boundaries.

Lastly, as in the case of the mass dimer, we observe again a scaled isospectrality of the dynamical matrices normalized with respect to the mid-gap frequency in strain and displacement coordinates for free boundaries as is shown in Supplementary Fig. **5**. Interestingly now, we have two additional zero modes for the case of displacement coordinates. This comes from the fact that the whole system can exhibit two types of rigid motion: A global translation and a global rotation.

D. Experimental implementation

1. Design

Beam modeling. To derive the structural stiffness of the beams, we consider a mechanical system consisting of two large cuboid blocks, playing the role of rigid particles, linked by two smaller cuboids in parallel (the beams), playing the role of the structural stiffness [see Supplementary Fig. **6(a)**]. The model assumes Euler-Bernoulli beam theory and linear (small) deformations. We described the lumped masses as being coupled by effective stiffnesses, denoted as K_c, where $c = B, S$ is the coupling index denoting bending and shear motions. We quantify these stiffnesses by analyzing the dumbbell system presented in Supplementary Fig. **6(a)** with different loading conditions on its end [6].

In our model, bending interaction induces only rotation (without translation) of the particles and can be derived for one of the two beams by relating the deflection ($\delta = \theta \cdot s/2$) of the beam with a bending moment [$M_b = F \cdot s/2$ with $F = (EA/L)\delta$ being the axial loading force] applied to one beam (the second block is assumed to be fixed). The final bending stiffness is found by changing the forces to torques by multiplying by the moment arm $d^2 = (W/2 + \frac{L}{2})^2$:

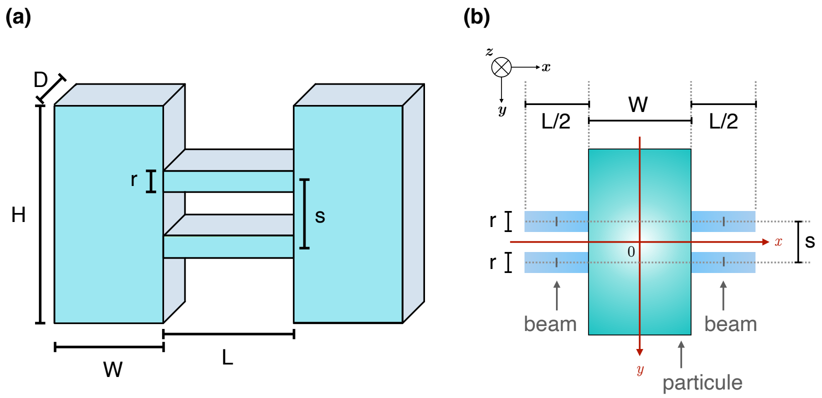

Supplementary Figure 6 | **(a)** Schematics of the mechanical system with notations. **(b)** Mechanical unit-cell.

$$K_{B1} = \frac{M_b}{\theta d^2} = \frac{EA}{Ld^2}\left(\frac{s}{2}\right)^2 \tag{27a}$$
$$= \frac{EDrs^2}{L(L+W)^2}, \tag{27b}$$

where s is the distance of separation between the center of the two parallel beams, L is the beam length, E is Young's modulus, A is the beam cross-section area, and subscript 1 in K_{B1} denotes that we are referring to one of two beams only.

Shear interaction involves only translation (without rotation) of the particles and can be defined by a combination of a shear load (P_s), and a bending moment (M_b) to enforce the boundary condition that $\theta(L) = 0$ (no rotation). The shear load induces a displacement denoted $\delta_s = P_S L^3/(3EI_b)$ and an angular deflection $\theta_s = P_S L^2/(2EI_b)$ of the beam, where I_b is the second moment of area of the beam. In addition, the bending moment will induce a displacement $\delta_b = M_b L^2/(2EI_b)$ and a angular deflection $\theta_b = M_b L/(EI_b)$ of the beam. To ensure that no rotation is obtained, we need to satisfy the relation $\theta_s = \theta_b$ which leads to the bending moment $M_b = P_S L/2$. The total displacement of the mass is then expressed as $u = \delta_s - \delta_b = P_S L^3/(12EI_b)$. Thus, the shear stiffness of one beam is obtained such as:

$$K_{S1} = \frac{P_S}{u} = \frac{12EI_b}{L^3}. \tag{28}$$

Finally, the equivalent effective springs for two beams in parallels can be obtained such that $K_c = 2K_{c1}$.

Therefore, the ratio of rigidity is expressed as:

$$\eta = \frac{K_B}{K_S} = \frac{L^2 s^2}{r^2(L+W)^2}. \tag{29}$$

Mass and moment of inertia calculations. The unit cell of the mechanical structure is composed of one large cuboid (the particle) and four smaller cuboids (the beams) as depicted in Supplementary Fig. 6**(b)**. In our model, we will consider that all the beams are identical. The total mass m and total moment of inertia I around the z-axis of the unit cell are defined as $m = m_\text{p} + 4m_\text{b}$ and $I = I_\text{p} + 4I_\text{b}$, where the index "p" and "b" refer to the particle and to the beam, respectively. For our cuboid system, the mass of the particle is expressed as $m_\text{p} = WDH\rho$, and the mass of one-half beam is $m_\text{b} = Dr(L/2)\rho$ where ρ is the density. The moment of inertia of the particle is given by $I_\text{p} = (1/12)m_\text{p}(H^2 + W^2)$. Applying the parallel axis theorem, we can obtain the moment of inertia of one half beam such as $I_\text{b} = (1/12)m_\text{b}(r^2 + (L/2)^2) + m_\text{b} d_1^2$, where $d_1 = \sqrt{(s/2)^2 + (W/2 + L/4)^2}$, which corresponds to the distance between the center of rotation of the particle and the center of the half beam. Note the difference in the order of magnitude of m_p and $4m_\text{b}$, meaning the mass of the four beams is small compared to the mass of the particle and could be neglected in the analysis for simplicity. The same approximation can be made for the moment of inertia.

Fine-tuned design. Our sample design is constrained by the fine-tuned relation: $P = 1/(1-\eta) = (md^2)/I$ where each term can be rewritten in terms of their geometrical parameters such as:

$$\frac{1}{1-\eta} = \frac{1}{1-\frac{L^2 s^2}{r^2(L+W)^2}}, \tag{30}$$

$$\frac{md^2}{I} = \frac{3D(2Lr+HW)(L+W)^2}{DHW(H^2+W^2)+2DLr\left[L^2+r^2+3LW+3(W^2+s^2)\right]}. \tag{31}$$

To design mechanical systems with different topologies (i.e. having different P values), our approach is the following. We first set all the geometric values except the parameters s and H corresponding to the distance of separation between the two centers of the beams and the height of the particle, respectively. For our system, the selected parameters are $W = 14$ mm, $D = 5$ mm, $L = 11$ mm, $r = 1.6$ mm. Then, imposing a value for P allows us to deduce the distance of separation by solving Eq. (30), which leads to $s = (r\sqrt{P-1}(L+W))/(L\sqrt{P})$. And finally, the last geometrical unknown is the height of the particle, which can be obtained by solving Eq. (31) seeking for H. For each structure, the height of the mass, H_i, and the distance of separation between the center of the two parallel beams, s_i, vary such as $H_A \simeq 22.4$ mm, $s_A \simeq 2.8$ mm, $H_B \simeq 26.8$ mm, $s_B \simeq 2.6$ mm and $H_C \simeq 32.5$ mm, $s_C \simeq 2.1$ mm for samples A, B, and C shown in Supplementary Fig. **3(b)**.

Finite element method simulations. To confirm the validity of our lumped-mass model, we obtain numerical dispersion curves via finite element methods (FEM) using COMSOL Multiphysics software, which are presented in Supplementary Fig. **3(c)**. These results are computed by modeling the unit cell in three dimensions shown in panel Supplementary Fig. **3(a)** and applying periodic boundary conditions on the sides of the two parallel beams. The following mechanical parameters are used for the material adopting a linear elastic constitutive law: density $\rho = 1180$ kg/m^3, Young's modulus $E = 2.74$ GPa, and Poisson ratio $\nu = 0.38$. Three-dimensional domains are meshed by means of three-dimensional 8-node hexahedral quadratic elements of maximum size $L_{FE} = 0.5$ mm, which is found to provide accurate eigensolutions up to the frequency of interest. The colormap reported in Supplementary Fig. **3(c)** describes the dominant component of the motion of the mass (such as $\frac{|u_f|}{|u_f|+|\phi_f|}$, where the index f denotes the mode index). It varies from pure rotation (yellow) to pure transverse displacement (dark blue).

2. Fabrication and measurements

Additive manufacturing of chains. The specimens, consisting of three classes of chains hosting 13 masses, are fabricated through additive manufacturing (Stratasys Objet350 Connex3). Thermoplastic polymer (VERO^{TM}), with the following nominal properties, has been used: density $\rho = 1180$ kg/m^3, Young's modulus $E = 2.74$ GPa, and Poisson ratio $\nu = 0.38$. The manufactured samples are presented in Supplementary Fig. **7(a)**.

Frequency response measurements. Supplementary Fig. **7(b)** presents the experimental spectra of each particle for the three tested samples having different values of P, namely $P = 2.5$, $P = 2$, and $P = 1.5$. Elastic waves are excited via an automatic modal hammer (Maul-Theet vImpact-61), striking the particle #2 (near the fixed end). The chains are suspended by hanging the particle #1 (clamped to ensure fixed boundary condition); therefore, all other particles are free. The experimental wave velocities are measured (and averaged over 10 repetitions) using a scanning Laser Doppler Vibrometer (SLDV) at three points on each particle, as presented in Supplementary Fig. **7(a)**. The laser sensitivity was set to 50 mm/s/V for all the measurements. In Supplementary Fig. **7(b)**, we present the experimentally measured spectra when the structure is excited near the fixed side, wherein the left panel shows the experimentally measured spectra for $P = 2.5$. The yellow color indicates the high amplitude of the spectra, and the black color means low amplitude. First, we can see that the particle #1 has a very low amplitude confirming that this particle is experimentally fixed. Second, particle #2 has a very high amplitude for all the frequency ranges presented, meaning that the impact excites a sufficient frequency range for our analysis. Looking now at all the other particles, in the range of frequency from 0 to 0.85, we observe a high amplitude of the spectra, which corresponds to the first band of propagation. From 0.85 to 1.15, we see that the wave is strongly decaying along the chain, which matches the predicted band gap (from 0.8 to 1.2). Then, between 1.25 to 1.4, the second band of propagation is measured. Similar conclusions can be made when $P = 1.5$. Considering $P = 2$, high amplitude exists from 0 to 1.4 without any band gap, as expected from our modeling. For the structure having $P = 1.5$, high energy is present in the band gap, a frequency around 1, which corresponds to the detection of a localized mode to the fixed side of the chain [see the right panel of Supplementary Fig. **7(b)**]. Supplementary Fig. **7(c)** presents the experimentally

measured spectra when the structure is excited from the free side (particle #13). The same conclusion can be made for the propagating and forbidden bands. However, now, for the structure having $P = 2.5$, high energy is present in the band gap at a frequency around 1, which corresponds to the localized mode at the free end of the chain [see left panel of Supplementary Fig. 7(c)].

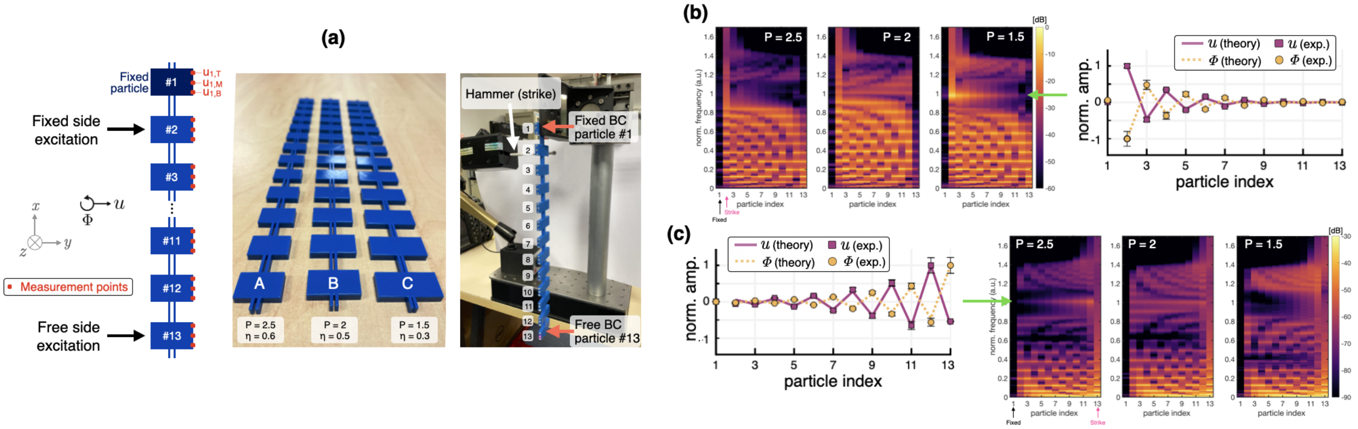

Supplementary Figure 7 | (a) Experimental setup configuration and manufactured samples. **(b)** Experimental spectra for each samples. Elastic waves are excited via an automatic hammer striking the particle #2 (near the fixed end). The associated localized mode at the fixed boundary is shown on the right panel. **(c)** Experimental spectra for each samples. Elastic waves are excited by striking the particle #13 (at the free end). The associated localized mode at the free boundary is shown on the left panel. Error bars denote variation (one standard deviation from averaged value) in experimental measurements.

Experiments vs simulations. We have conducted frequency response calculations (Bode calculation via Matlab software) of our systems when exciting them near the fixed or free ends. The comparison between the frequency response calculations and the experimental results is presented in Supplementary Fig. 8. These calculations are carried out for chains of 12 particles (excluding the fixed particle) with fixed-free boundary conditions. In addition, we consider dissipation via a "Kelvin-Voigt" model where the viscous damping is defined by a decay time of the wave, which is $\tau = 18$ ms in our case. First, we observe a good agreement between the experimental results and the frequency response calculations. The propagating and forbidden bands are recovered, as well as the two localized modes at the fixed or free edges depicted by the two green arrows.

Decomposing the transverse and rotational components. Complex velocities, in terms of amplitude and phase, are measured on three different points on each particle. We denote, $u_{n,\mathrm{T}}$, $u_{n,\mathrm{M}}$, and $u_{n,\mathrm{B}}$, the measurement point at the top, at the middle and at the bottom of the nth particle, as depicted in Supplementary Fig. 7(a). We assume a small displacement of the particle. The transverse motion u_n and the rotation ϕ_n can be decomposed via trigonometric algebra and are given as:

$$u_n = S_{n,M}|u_{n,\mathrm{M}}|, \tag{32a}$$

$$\phi_n = \frac{S_n}{2}\left[\arctan\left(\frac{|u_{n,\mathrm{T}}| - |u_{n,\mathrm{M}}|}{h_\mathrm{T}}\right) + \arctan\left(\frac{|u_{n,\mathrm{B}}| - |u_{n,\mathrm{M}}|}{h_\mathrm{B}}\right)\right], \tag{32b}$$

where $S_{n,M} = \mathrm{sign}\left(\Re[u_{n,\mathrm{M}}]\right)$ and takes 1 or -1 value, and $Sn = -1$ if $\Re[u_{n,\mathrm{T}}] > \Re[u_{n,\mathrm{B}}]$ or $S_n = 1$ if $\Re[u_{n,\mathrm{T}}] < \Re[u_{n,\mathrm{B}}]$. The variables h_T and h_B correspond to the distances between the top and middle point of measurement and from the bottom to the middle point of measurement, respectively (in our case $h_\mathrm{T} = h_\mathrm{B} = 6$ mm). From this mode decomposition, we are able to reconstruct the mode shape at a given frequency, such as is presented in Supplementary Figs. 7(b-c). Finally, Supplementary Fig. 9(c) shows the component amplitudes as a function of the frequency from the measured data and from the analytical prediction for the three structures.

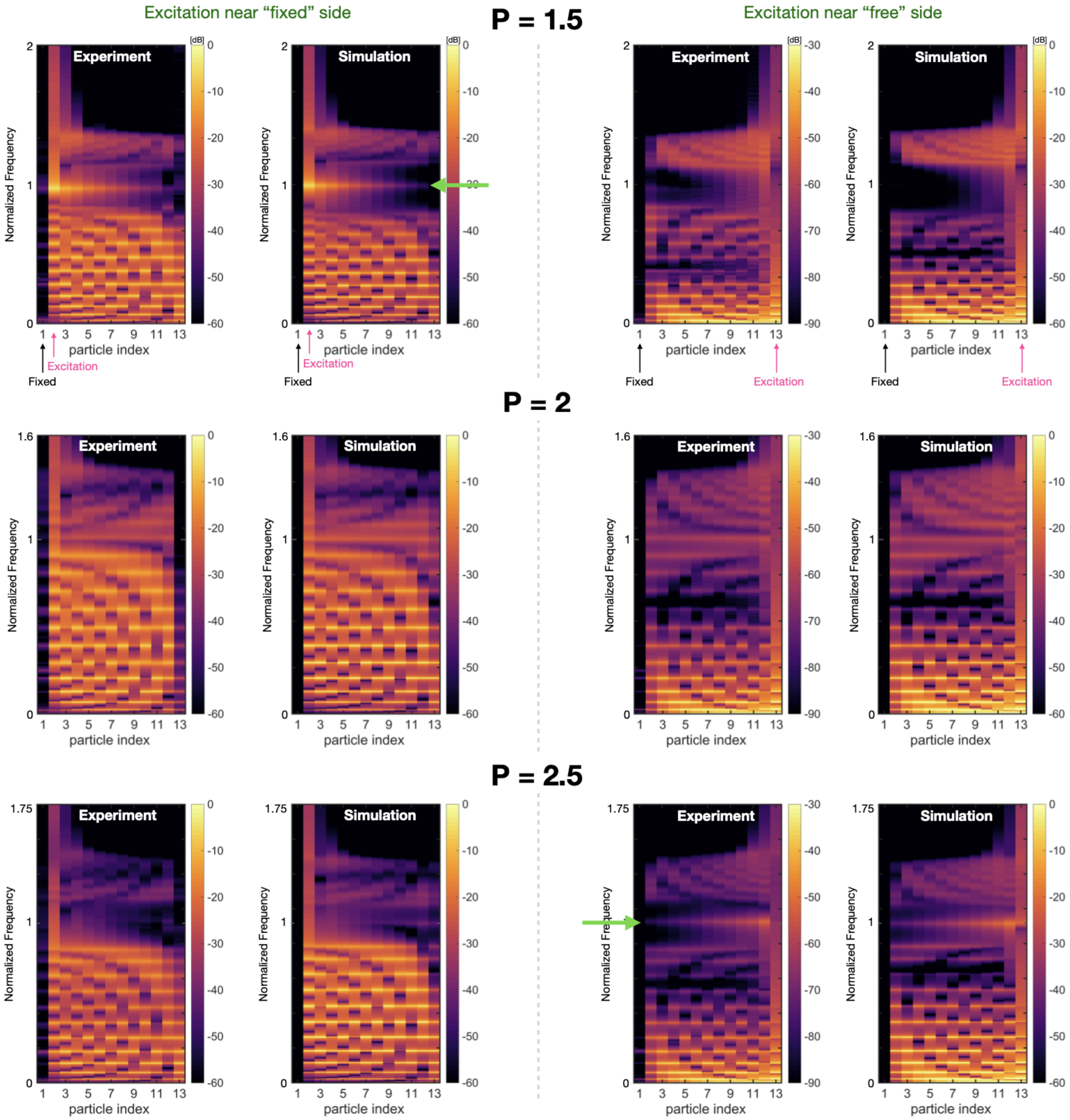

Supplementary Figure 8 | Experimental spectra versus frequency response calculations (Bode calculation) using Kelvin-Voigt model for the dissipation (decay time of the wave is $\tau = 18$ ms). The two green arrows show the two localized modes at the fixed or free edges.

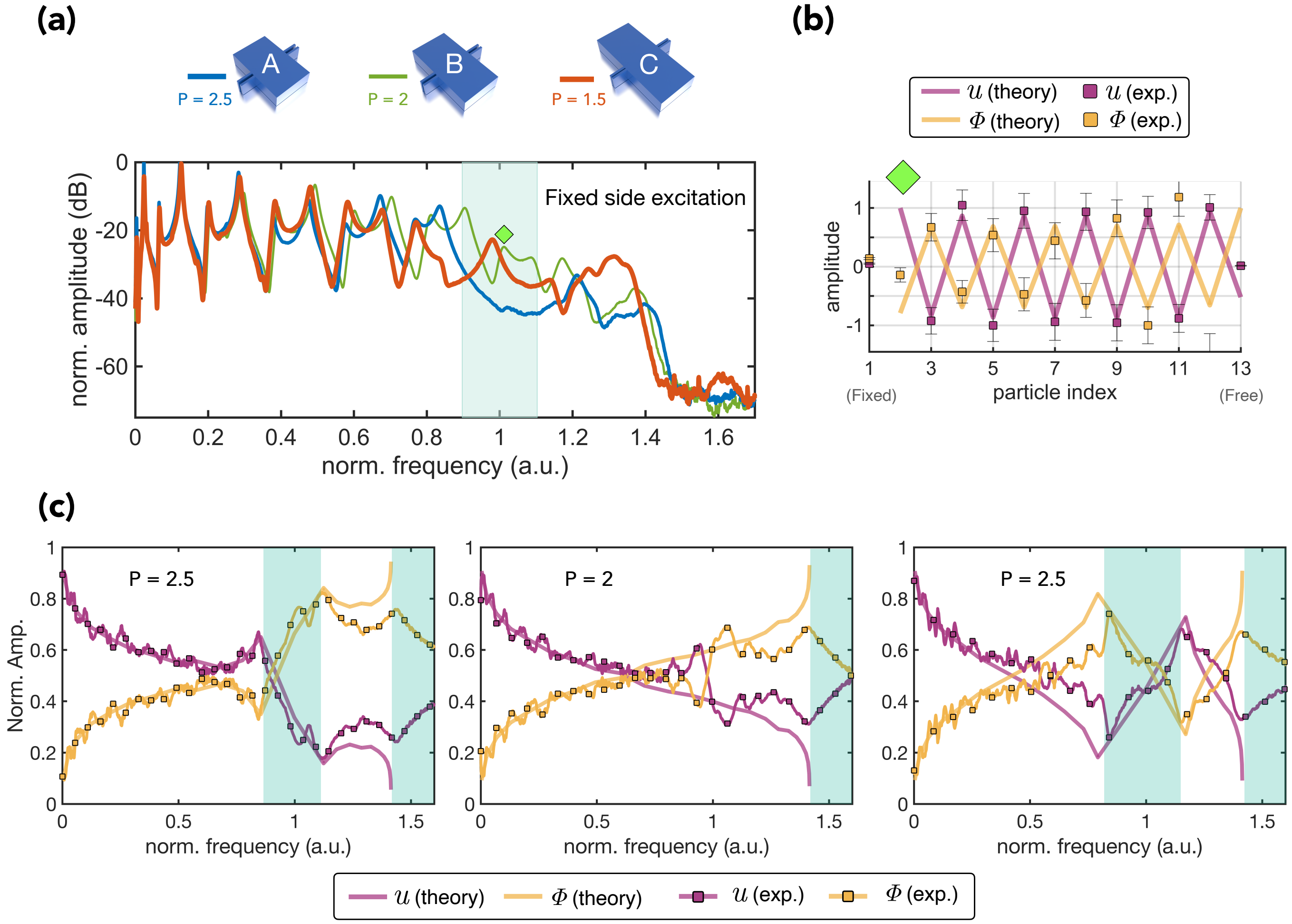

Supplementary Figure 9 | **(a)** Experimental spectra from particle #7 measured at the middle of the particle (transverse motion) for excitation near the fixed end (particle #2). The blue area corresponds to the band gap prediction. **(b)** Mode shape obtained in the structure B (P = 2), corresponding to the frequency marked by the green diamond in panel **(a)**. The mode is no longer localized to a boundary but rather extends over the entire chain confirming the band gap closing at P = 2. Error bars denote variation (one standard deviation from averaged value) in experimental measurements. **(c)** Component amplitudes (u_y vs. ϕ) as a function of the normalized frequency for each structure.

III. SUPPLEMENTARY NOTE 3 – DISORDER ANALYSIS

Another implication of topology is the robustness of edge states against disorder. Especially for symmetry-preserving disorders in the chain, the topological edge states are extremely robust. We will provide here evidence that finite-frequency topological mechanical metamaterials, both normal and strain, support edge states that have the same level of protection against various kinds of disorders. For the case of strain topological metamaterials, the disorder analysis is applied in strain coordinates, where, in the corresponding strain dynamical matrices, the symmetry-preserving disorder becomes apparent.

A. Mass vs. stiffness dimer

First, to check the robustness of the edge state in the mass dimer, we present a generalized spring-mass system that could be reduced to either the mass dimer or the stiffness dimer (finite-frequency mechanical SSH chain). The *displacement* dynamical matrix for an $2N$-particle chain, *fixed* at both ends, has the following tridiagonal form:

$$\hat{D}_u = \begin{bmatrix} \frac{k_1+k_2}{m_1} & -\frac{k_2}{\sqrt{m_1}\sqrt{m_2}} & \dots & 0 & 0 \\ -\frac{k_2}{\sqrt{m_1}\sqrt{m_2}} & \frac{k_2+k_3}{m_2} & -\frac{k_3}{\sqrt{m_2}\sqrt{m_3}} & \dots & 0 \\ \dots & \dots & \dots & \dots & \dots \\ 0 & \dots & -\frac{k_{2N-1}}{\sqrt{m_{2N-2}}\sqrt{m_{2N-1}}} & \frac{k_{2N-1}+k_{2N}}{m_{2N-1}} & -\frac{k_{2N}}{\sqrt{m_{2N-1}}\sqrt{m_{2N}}} \\ 0 & 0 & \dots & -\frac{k_{2N}}{\sqrt{m_{2N-1}}\sqrt{m_{2N}}} & \frac{k_{2N}+k_{2N+1}}{m_{2N}} \end{bmatrix}_{2N\times 2N}. \quad (33)$$

Similarly, the *strain* dynamical matrix for an $(2N+1)$-particle chain, *free* at both ends (i.e., $k_1 = k_{2N+2} = 0$), has the following tridiagonal form:

$$\hat{D}_s = \begin{bmatrix} k_2(\frac{1}{m_1}+\frac{1}{m_2}) & -\frac{\sqrt{k_2}\sqrt{k_3}}{m_2} & \dots & 0 & 0 \\ -\frac{\sqrt{k_2}\sqrt{k_3}}{m_2} & k_3(\frac{1}{m_2}+\frac{1}{m_3}) & -\frac{\sqrt{k_3}\sqrt{k_4}}{m_3} & \dots & 0 \\ \dots & \dots & \dots & \dots & \dots \\ 0 & \dots & -\frac{\sqrt{k_{2N-1}}\sqrt{k_{2N}}}{m_{2N-1}} & k_{2N}(\frac{1}{m_{2N-1}}+\frac{1}{m_{2N}}) & -\frac{\sqrt{k_{2N}}\sqrt{k_{2N+1}}}{m_{2N}} \\ 0 & 0 & \dots & -\frac{\sqrt{k_{2N}}\sqrt{k_{2N+1}}}{m_{2N}} & k_{2N+1}(\frac{1}{m_{2N}}+\frac{1}{m_{2N+1}}) \end{bmatrix}_{2N\times 2N}. \quad (34)$$

We start with the classical case of the stiffness dimer chain, and several types of disorders, and compare their behavior to that in the mass dimer. It is easy to see that the displacement dynamical matrix in Eq. (33) represents the stiffness dimer when $m_i = m$ for $i \in [1, 2N]$, and $k_{2i-1} = k_1 = k_{2N+1}$, $k_{2i} = k_2$ for $i \in [1, N]$. The reduced matrix, therefore, has a constant diagonal ($\frac{k_1+k_2}{m}$), which can be removed, through subtraction of a multiple of the identity matrix, and the remaining matrix is chiral. Similarly, the strain dynamical matrix in Eq. (34) represents the mass dimer when $k_i = k$ for $i \in [1, 2N]$, and $m_{2i-1} = m_1 = m_{2N+1}$, $m_{2i} = m_2$ for $i \in [1, N]$. Again, the reduced matrix has a constant diagonal $[k(\frac{1}{m_1}+\frac{1}{m_2})]$, which can be removed as in the previous case, and the remaining matrix is chiral. We are now ready to introduce several types of disorders in the mass and stiffness dimer chains noting that the effect of disorder on the diagonal terms would be crucial in the topological protection of edge states.

Disorder with no symmetry: In stiffness dimer, we introduce random disorder such that the stiffnesses in the nth unit cell change to $k_{1,n} = k_1 + \gamma\lambda_n$ and $k_{2,n} = k_2 + \gamma\phi_n$, where γ is the disorder strength and λ_n and ϕ_n are random numbers that follow a uniform distribution in $[-1, 1]$. Similarly, in the mass dimer, we vary masses as $m_{1,n} = m_1 + \gamma\lambda_n$, $m_{2,n} = m_2 + \gamma\phi_n$. The initial masses (of the clean case) were chosen such that $P = \frac{m_1}{m_2} = 0.4$, as the experimental sample of mass dimer. For the stiffness dimer we use $\eta = \frac{k_2}{k_1} = 0.4$. In experimentally realized mechanical systems, such as those we present in this work, the mass disorder appears due to modification of the size or density of the particles, while stiffness disorder occurs due to modifications of the beams connecting the particles. In this disorder analysis, we ensure that the disorder strength is experimentally achievable, and we also ensure the stability of the systems by considering only positive quantities for the stiffnesses and masses ($\gamma < 1$). In Supplementary Fig. **10(a)**, we show the effect of this disorder on the spectrum of stiffness and mass dimers. We present the averaged spectrum of 1000 realizations of disorder for 50 and 51 particle-chains in the case of the stiffness and mass dimers, respectively. We observe the spectra of both the stiffness and mass dimers lose their symmetry about the mid-gap

frequency ($\omega^2 = 1$). Particularly, the edge states (localized on both ends) inside the band gap deviate from the midgap frequency. This is because of the loss of chiral symmetry of the finite dynamical matrix after introducing disorder. For the stiffness and mass dimers, the diagonal terms in Eq. (33) and Eq. (34) are no longer constant under such disorder, and therefore, it is not possible to restore chiral symmetry after substraction of a multiple of the identity matrix.

Disorder with inversion symmetry: Next, we introduce disorder in mass and stiffness dimer chains such that the respective disorders are symmetric about the center of the finite chains. In Supplementary Fig. **10(b)**, we show the averaged spectrum for both stiffness and mass dimer under this disorder. Again, we observe that the spectrum loses its chirality due to this disorder. However, we note the partial protection of the edge states in *both* the mass and stiffness dimers. The two edge states, localized at opposite ends, are not exactly pinned at the mid-gap frequency, but they don't split due to the inversion symmetry of disorder in the finite chains.

Disorder with chiral symmetry: What disorder keeps the chiral symmetry of the stiffness and mass dimer chain? We consider the stiffness dimer chain first. There are two ways to introduce chiral disorder in this chain. The first way is through introducing disorder solely on stiffness, and the second way is also to alter the masses. The first way was introduced recently [2], where the chiral symmetry of the dynamical matrix was preserved by introducing a ground stiffness for each mass. This leads to the addition of an onsite stiffness in the diagonal elements of Eq. (33). Disorder in k can then be balanced by the disorder in onsite stiffness, to keep the diagonal constant and the matrix chiral.

We present the second way here based on engineered variations of the masses avoiding the introduction of ground stiffnesses. It is clear from Eq. (33) that the diagonal elements $\eta = \frac{k_i + k_{i+1}}{m_i}$ can be kept constant if the disorder in k terms is balanced by disorder in m terms. We allow $m_i = m + \rho_i$, where m is the initial mass (same for all the particles in the case of the stiffness dimer) and fine-tune ρ_i, such that: $\rho_i = \frac{k_i + k_{i+1}}{\eta} - m$. The same procedure can then be translated to the stain dynamical matrix for the mass dimer in Eq. (34) as well. We keep the diagonal elements constant, such that $P = k_i(\frac{1}{m_{i-1}} + \frac{1}{m_i})$ by varying $k_i = k + \tau_i$ with $\tau_i = P/(\frac{1}{m_{i-1}} + \frac{1}{m_i}) - k$, where k is the initial stiffness. In Supplementary Fig. **10(c)**, we show the spectra of mass and stiffness dimer under chiral disorder. It is evident that this disorder keeps the symmetry of the spectrum about the midgap frequency. The edge states are extremely robust under such disorder. We have also verified that the chiral signature is present in the edge states where their amplitudes vanish at alternating sites (bonds) for the stiffness dimer (mass dimer) when $\gamma = 1$.

Therefore, we have demonstrated that the mass dimer (strain topological chain) supports edge states that are topologically protected against chiral disorder, while they also keep a high level of protection under disorder that respects the inversion symmetry. This provides extra evidence that strain topological metamaterials *are* topological, and their topology (bulk index and protecting symmetries) can only be uncovered in strain coordinates.

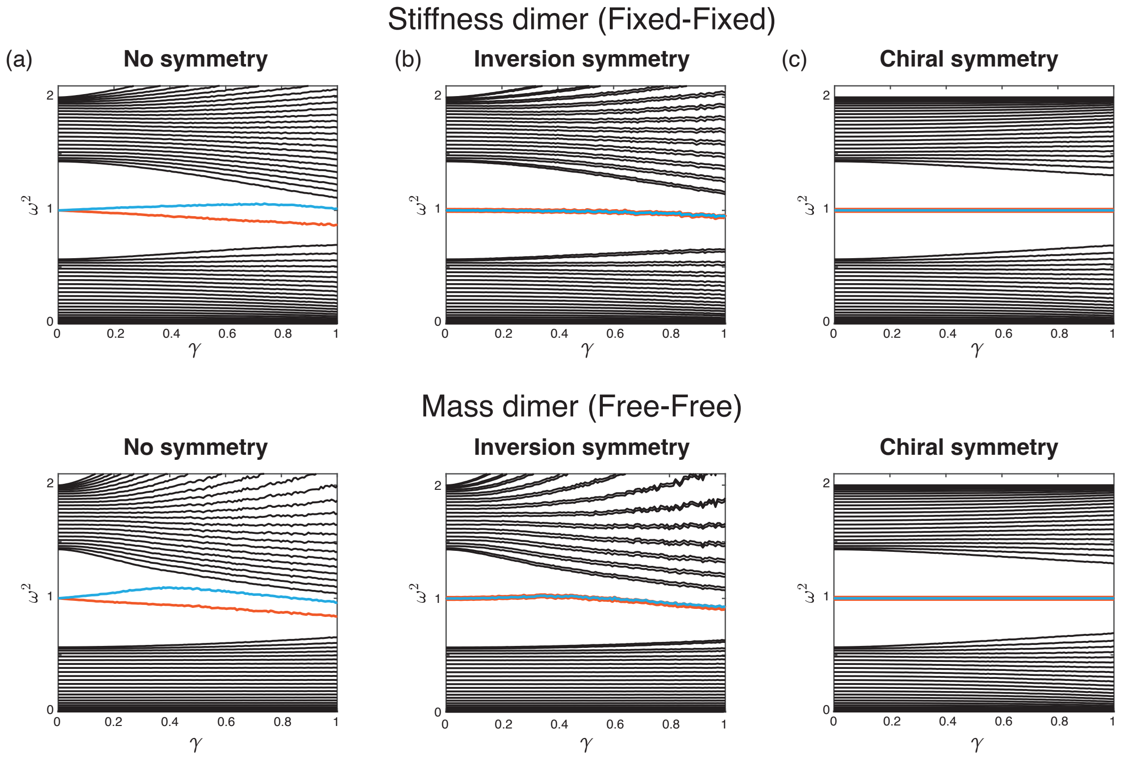

Supplementary Figure 10 | Disorder analysis of the stiffness and mass dimer chains. Spectra of the stiffness dimer (top row) and mass dimer (bottom row) as a function of the disorder strength γ when (a) no symmetry, (b) inversion symmetry, and (c) chiral symmetry are preserved. Left and right localized edge states are denoted in red and blue.

B. Mechanical Kitaev chain

The mechanical Kitaev chain supports topological edge states at both fixed and free ends. However, the topological origin of the latter is revealed in the strain coordinates. In this section, we will show the edge states for fixed-fixed and free-free chains have equivalent robustness against various types of disorders.

The equations of motion for the mechanical Kitaev chain are:

$$\begin{aligned}\ddot{u}_n &= \frac{K_{S,n}}{m_n}\left(u_{n-1}-u_n\right)-\frac{K_{S,n+1}}{m_n}\left(u_n-u_{n+1}\right)\\ &\quad+\frac{K_{S,n}}{m_n}\left(\Phi_n+\Phi_{n-1}\right)-\frac{K_{S,n+1}}{m_n}\left(\Phi_n+\Phi_{n+1}\right), \qquad (35a)\\ \ddot{\Phi}_n &= P_n\frac{K_{S,n}}{m_n}\left(u_n-u_{n-1}-\left(\Phi_{n-1}+\Phi_n\right)\right)\\ &\quad+P_n\frac{K_{S,n+1}}{m_n}\left(u_{n+1}-u_n-\left(\Phi_n+\Phi_{n+1}\right)\right)\\ &\quad+P_n\frac{K_{B,n}}{m_n}\left(\Phi_{n-1}-\Phi_n\right)-P_n\frac{K_{B,n+1}}{m_n}\left(\Phi_n-\Phi_{n+1}\right), \qquad (35b)\end{aligned}$$

where $P_n=\frac{m_n d_n^2}{I_n}$. We introduce the following types of disorders:

Disorder with no symmetry: We implement disorder without symmetry in two ways. First, we apply a disorder in the mass/inertia (leading to a disorder in values of P_n), and second, apply a disorder in K_B (leading to a disorder in the values of $\eta_n = K_{B,n}/K_S$). The first type of disorder is implemented by inducing a disorder in the height of each particle (H_n), while the second disorder is created by changing the distance between the two beams (s_n). The disorders are constrained to realistic physical quantities (ensuring a possible construction of these structures). The initial conditions, are: $N_{fixed} = 30$ particles with $P = 1.5$, and $N_{free} = 31$ particles with $P \to P/(P-1)$. We have

applied disorder on the mechanical beam in the following manner: $s_n = s_s + s_s\gamma\lambda_n$, where s_s is the starting value without disorder of the distance between the two beams, γ is the disorder strength, and λ_n is a uniform random number in $[-1, 1]$. In an analogous way, the particle height is modified such as $H_n = H_s + H_s\gamma\lambda_n$, where H_s is the starting value without disorder of the particle height. Spectra of the mechanical chain as a function of disorder strength are presented in Supplementary Fig. **11**. In Supplementary Figs. **11(a,c)**, we observe that the edge states are no longer "pinned" at the mid-gap frequency, and they "split" as the disorder strength grows. This is expected due to a random disorder in P and η. However, the degree of protection is seemingly close for the edge states in fixed-fixed and free-free chains.

Disorder with inversion symmetry: We again introduce disorder separately in P and η as before, but now keep the inversion symmetry of the finite disordered chain intact. In Supplementary Figs. **11(b,d)**, the edge states are now partially protected as they do not split. We observe that inversion symmetry adds an "extra" protection to both fixed-fixed and free-free boundary conditions equally.

Disorder with particle-hole symmetry: Since our mechanical Kitaev chain possess particle-hole symmetry after fine-tuning (as shown in the main text), we look to introduce disorder in this system that preserves this symmetry. Note that since the dynamical matrices are real, particle-hole and chiral symmetry coincide. However, there is no physical way to apply such a disorder in the current mechanical design, without adding additional elements. This is because we have already imposed a constraint ($\eta = 1 - 1/P$) between effective stiffnesses and masses for fine-tuning. Thus we do not have the same freedom for extra fine-tuning to preserve particle-hole as in the case of the mass-stiffness dimer. Since there exists no disorder that preserves the particle-hole (equivalently chiral) symmetry of the fixed-fixed Kitaev chain (in displacement coordinates) in the current mechanical design, it also implies the absence of such disorder in the free-free chain (in strain coordinates). However, as suggested, there could be other ways to introduce disorder that preserves particle-hole symmetry, such as modifying the current mechanical design by adding extra elements, as was done for the stiffness dimer in [2].

Therefore, we have demonstrated that the mechanical Kitaev chain (under the current mechanical design constraints) supports topological edge states that are robust against disorders that respect inversion symmetry. Importantly, we have shown that the edge states at free ends (revealed in strain coordinates) have a similar degree of protection as compared to the edge state at fixed ends (revealed in displacement coordinates).

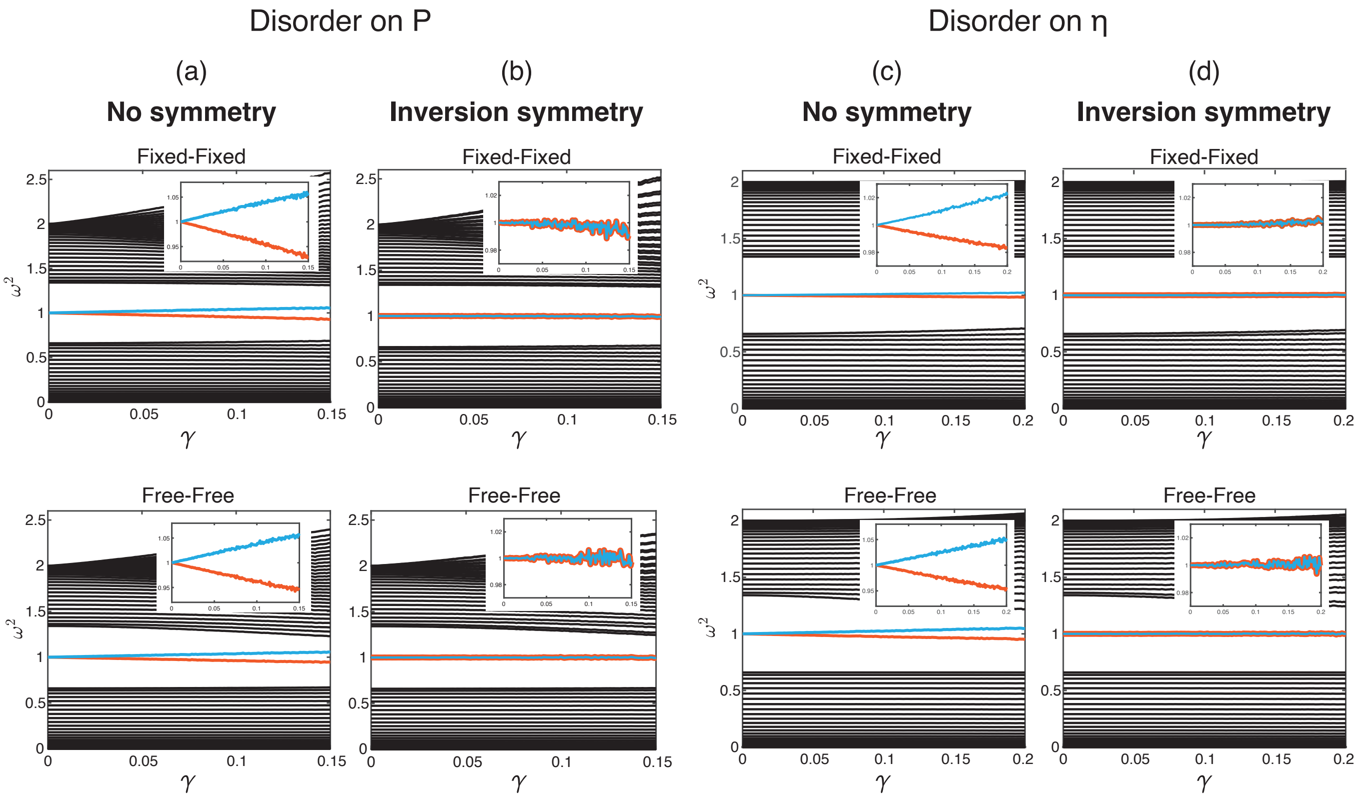

Supplementary Figure 11 | Disorder analysis - Kitaev chain. Spectra of the mechanical Kitaev chain as a function of the disorder strength γ for **(a, b)** disorder on P (mass/inertia) or **(c, d)** disorder on η (stiffness). Top line and bottom line refer to fixed-fixed and free-free boundary conditions, respectively. Panels **(a, c)** display the results when no symmetry is present, while panels **(b, d)** show the results with inversion symmetry conserved. The insets correspond to a focus on the topological edge state's frequency. The results are averaged over 1000 iterations for each disorder strength.

IV. SUPPLEMENTARY NOTE 4 – PHOTONIC ANALOGIES TO MASS AND SPRING MODELS

Here we show a class of photonic systems for which the transverse-electric polarised light dynamics can map to those of a mass and spring model within certain parameter regimes, as demonstrated in Ref. [7]. The photonic system is composed of a series of voids of half height $H_{1,2}$ connected by thin channels of half height $h_{1,2}$, as shown in Supplementary Fig. **12(a)**. Each unit cell for the dimer systems is composed of two voids and two channels and has unit cell spacing $a = 2L$. The boundaries of the voids are composed of a perfect electrical conductor and the areas are vacuum. As shown in [7] the mapped masses are proportional to the area of the voids, such that:

$$m_i = A_i \cdot m_0/L^2, \tag{36}$$

where A_i is the area of the ith void and m_0 is defined in terms of the speed of light c_0 such that $c_0 = \sqrt{k_0/m_0}$. The mapped spring constants are defined as

$$k_i = \frac{1}{\pi}\sqrt{\frac{2h_i}{R_i}} \cdot k_0, \tag{37}$$

where R_i is the radius of curvature of the side of the channel and k_0 is defined similarly to m_0 in terms of c_0.

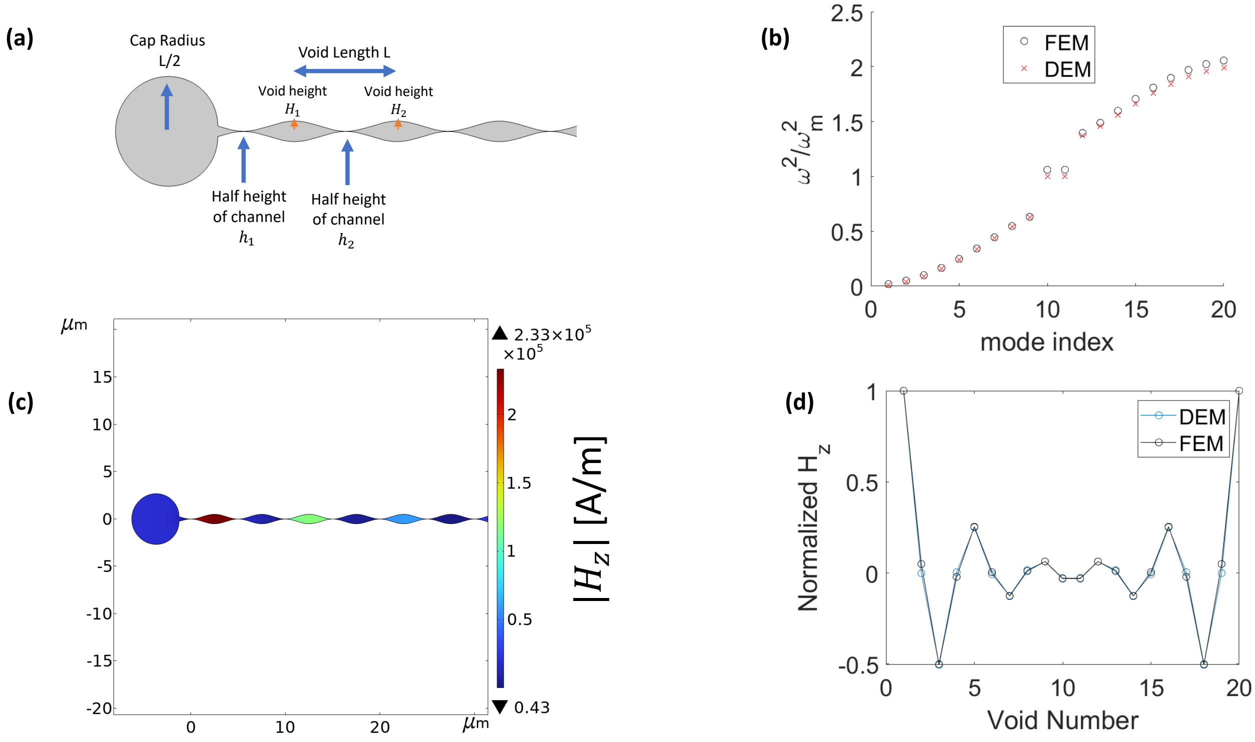

Supplementary Figure 12 | Photonic SSH mass and spring chain analog. **(a)** Geometry and relevant parameters. **(b)** Spectrum normalized to the analytical center frequency $\omega_m^2 = (k_1 + k_2)/m$ calculated using both FEM and DEM. **(c)** The topological edge mode visualized in terms of the out-of-plane magnetic field H_z. A topological edge mode (mode index 10) calculated both using FEM and DEM. **(d)** A topological edge mode (mode index 10) calculated both using FEM and DEM.

A. Photonic Su-Schrieffer-Heeger (SSH) chain

A photonic SSH chain is modeled by setting the heights of each void to be the same ($H_1 = H_2 = H$). For a topological SSH chain the intra-cell coupling needs to be greater than the inter-cell hopping, *i.e.* $k_2 > k_1$. Here, we use same parameters as in Ref. [7] ($H = L/10$, $h_1 = H/400$, $h_2 = H/100$) and set $L = 5$ μm to create a $N = 20$

particle chain. Both ends of the chain are capped with circular voids of radius $L/2$ to provide "fixed" boundary conditions. The geometry of the chain is given by the function:

$$y(x) = \pm[H \sin(\pi x/L)^2 + h_1 \sin(\pi x/(2L))^2 + h_2 \cos(\pi x/(2L))^2], \tag{38}$$

where $x \in [-L/4, (N+1/4)L]$. For this case, the $R_i = R = L^2/(2\pi^2 H)$ and $A_i = A = HL$, such that the bulk of the chain is composed of identical masses m and alternating springs with stiffness k_1 and k_2. The left and right end caps are given respectively by:

$$X_L(\theta) = \frac{L}{2}\cos(\theta) - \frac{3}{4}L, \tag{39}$$

$$Y_L(\theta) = \frac{L}{2}\sin(\theta) + h_{end}\cos(\theta/2), \tag{40}$$

$$X_R(\theta) = \frac{L}{2}\cos(\theta) + (N + \frac{3}{4})L, \tag{41}$$

$$Y_R(\theta) = \frac{L}{2}\sin(\theta) - h_{end}\sin(\theta/2), \tag{42}$$

with $h_{end} = y(-L/4) = y(N+1/4)L$. The resulting geometry is shown in Supplementary Fig. **12(a)**. The finite spectrum and normal modes are calculated with both FEM using COMSOL Multiphysics v6.0 and a discrete element model (DEM) using MATLAB R2021a, and are shown in Supplementary Figs. **12(b-d)**.

B. Photonic mass dimer

A photonic equivalent of a mass dimer system is modeled by setting $p = m_1/m_2 = 0.25$. We set the first mass to have height $H_1 = L/20$, and the second height as scalar multiple times the first height $H_2 = \alpha H_1$, where the chain geometry is given by the function:

$$y(x) = \pm[((H_1 + H_2)/2 + ((H_1 - H_2)/2)\sin(\pi x/L))\sin(\pi x/L)^2 + h], \tag{43}$$

noting that L is set to be the same as in the SSH example and $h = h_1 = h_2 = H_1/400$. The height scaling factor α is found by integrating Eq. (43) to achieve the desired mass ratio, such that $\alpha = 5.8226$. This results in a chain where the bulk of the chain is composed of alternating masses m_1 and m_2 and identical springs with stiffness $k = k_1 = k_2$, as defined in Eq. (37). For calculating the stiffness k, as in Eq. (37), we define the radius of curvature of the channel to be:

$$R = \left| \frac{(1 + \frac{dy}{dx})^{3/2}}{\frac{d^2y}{dx^2}} \right| = \frac{L^2}{\pi^2(H_1 + H_2)}, \tag{44}$$

where $y(x)$ is given by Eq. (43). Free boundary conditions are implemented by cutting the geometry at the edge of a unit cell and not including end caps. The system parameters were chosen so that it maps to the mass dimer system considered in the experimental section of the main text for $P = 0.25$, $N = 7$. The resulting geometry is shown in Supplementary Fig. **13(a)**. The finite spectrum and normal modes are again calculated with both FEM and DEM, shown in Supplementary Figs. **13(b-d)**.

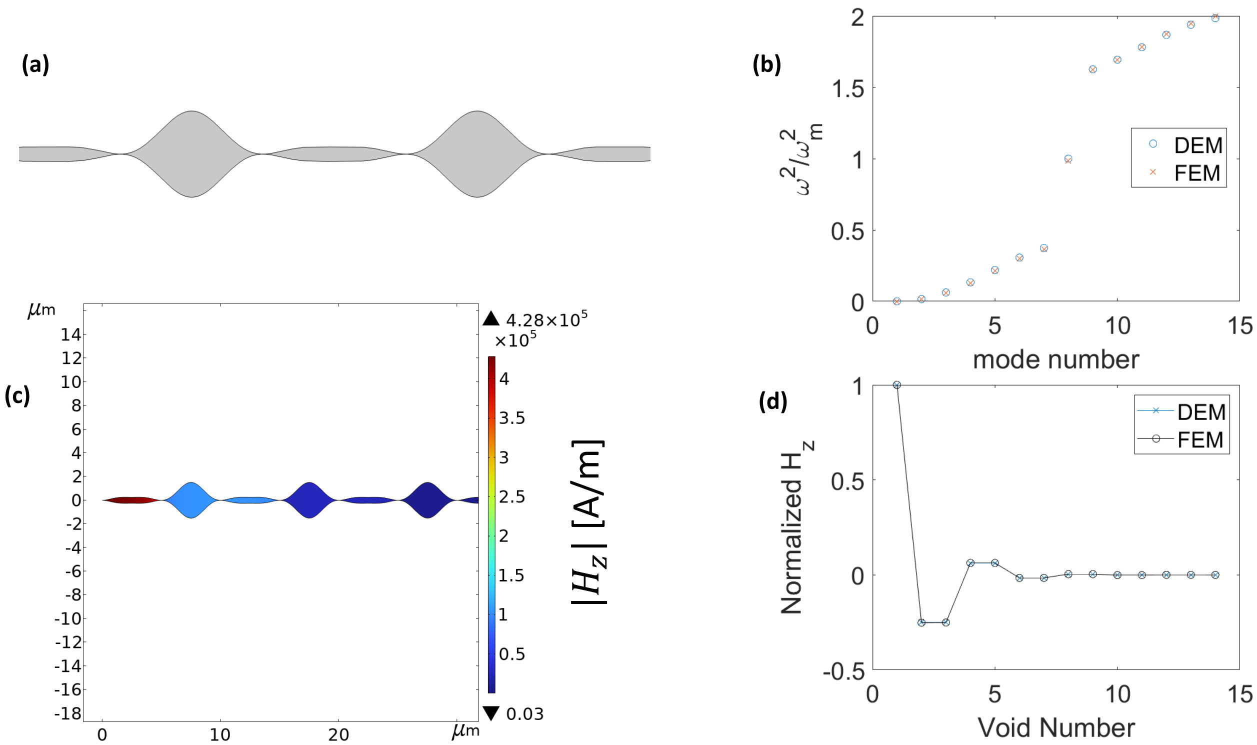

Supplementary Figure 13 | Photonic mass dimer analog equivalent to the experimental system studied in the main text. **(a)** Geometry. **(b)** Spectrum calculated through both DEM and FEM for the photonic mass dimer normalized by $\omega_m^2 = k(1/m_1 + 1/m_2)$. **(c)** Topological edge mode for mass dimer system (mode index 7). **(d)** Calculated topological edge modes from both FEM and DEM (mode index 7) and normalized to the largest amplitude field component H_z.

[1] W. P. Su, J. R. Schrieffer, and A. J. Heeger, Solitons in polyacetylene, Phys. Rev. Lett. **42**, 1698 (1979).
[2] X. Shi, I. Kiorpelidis, R. Chaunsali, V. Achilleos, G. Theocharis, and J. Yang, Disorder-induced topological phase transition in a one-dimensional mechanical system, Phys. Rev. Research **3**, 033012 (2021).
[3] A. Suiker, A. Metrikine, and R. De Borst, Dynamic behaviour of a layer of discrete particles, part 1: Analysis of body waves and eigenmodes, Journal of Sound and Vibration **240**, 1 (2001).
[4] L.-Y. Zheng, H. Pichard, V. Tournat, G. Theocharis, and V. Gusev, Zero-frequency and slow elastic modes in phononic monolayer granular membranes, Ultrasonics **69**, 201 (2016).
[5] N. Leumer, M. Marganska, B. Muralidharan, and M. Grifoni, Exact eigenvectors and eigenvalues of the finite kitaev chain and its topological properties, J. Phys. Condens. Matter **32**, 445502 (2020).
[6] J. Gere, Mechanics of Materials, Sixth Edition (Thomson ed., London, 2004).
[7] S. Palmer, Y. Ignatov, R. Craster, and M. Makwana, Asymptotically exact photonic approximations of chiral symmetric topological tight-binding models, New Journal of Physics **24**, 053020 (2022).
